# CFL1 is Implicated in Chronic Myeloid Leukemia Response during Imatinib Therapy

**DOI:** 10.7150/jca.92202

**Published:** 2024-03-04

**Authors:** Xiufeng Yin, Xia Li, Hao Jiang, Xiangjie Lin, Zhixin Ma, Xiaochang Chen, Qibei Teng, Jin Zhang, Jie Jin

**Affiliations:** 1 Department of Hematology, Sir Run Run Shaw Hospital, Zhejiang University School of Medicine, Hangzhou, P R China.; 2 Department of Hematology, The First Affiliated Hospital, Zhejiang University School of Medicine, Hangzhou, P R China.; 3 Department of Laboratorial Medicine, Women's Hospital, School of Medicine, Zhejiang University, Hangzhou, P R China.; 4 Clinical Prenatal Diagnosis Center, Women's Hospital, School of Medicine, Zhejiang University, Hangzhou, P R China.; 5 Zhejiang Provincial Key Lab of Hematopoietic Malignancy, Zhejiang University, Hangzhou, P R China.

**Keywords:** Chronic myeloid leukemia, Prognostic value, Imatinib mesylate, CFL1, F-actin

## Abstract

Cofilin (CFL1) is one critical member of the actin deploy family (ADF). Overexpression of CFL1 is associated with aggressive features and poor prognosis in malignancies. We evaluated the expression of CFL1 in patients with chronic myeloid leukemia in the chronic phase (CML-CP), acute myelocytic leukemia (AML) and healthy controls. The role of CFL1 in imatinib therapy was also investigated using cell line. We found that the expression of CFL1 was lower in CML patients than that in healthy controls, and was significantly upregulated after imatinib therapy (p<0.05). CML patients with lower CFL1 achieved higher Major molecular response (MMR) rate after 6 months of imatinib therapy (p<0.05). Cofilin, P-cofilin and F-actin, especially branched F-actin were all upregulated after imatinib therapy. The lower CFL1 expression before treatment may predicts a better response to imatinib. Imatinib affects F-actin remodeling in CML patients by regulating CFL1 expression and activity.

## Introduction

After the introduction of imatinib as a new agent for this disease, the treatment of CML has undergone a revolution. In patients with chronic myeloid leukemia in the chronic phase (CML-CP), the 10-year survival rate has improved from less than 15% to more than 90% by imatinib 1-3, which has been approved as the first-line therapy or CML since 2003. CML patients are monitored carefully and benefit from early intervention and modifying therapy 4. It is well known that BCR/ABL transcriptional level ≤ 10% is one of the independent predictors for long-term survival 5,6. These predictors can guide physicians to modify treatment regimens as early as possible for a better outcome for these patients 7. Evidence showed that patients who failed to respond to imatinib in the early stages and were given second-generation TKIs achieved a durable response, with approximately 80% surviving after a 4-year follow-up 8. Therefore, it is critical to identify such patients early.

BCR-ABL, the oncogene of CML, exerts a variety of biological activities, including cell proliferation, anti-apoptosis, cell migration, etc. 9. Apart from constitutively activated Tyrosine Kinase caused by SH1 and SH2 function domain, it contains an actin-binding domain in C-terminal which influences cell adherence and migration via the F-actin function, mediated by integrin signal pathway 10. Actin-binding domain is involved in leukemia development in vivo 11. It is known that a large number of immature myeloid cells released into the peripheral blood is caused by decreased adhesion of CML cells to the extracellular matrix 12. The altered adhesion is independent of BCR-ABL kinase activity 13. Cofilin is a critical regulator of the actin filament (F-actin) assembly or disassembly by severing or stabilizing F-actin 14. Is there any relationship between CML and cofilin? What role does it play during Imatinib therapy? Here, we investigate cofilin expression in CML to address these concerns.

## Material and Methods

### Patients and therapy

Inclusion criteria were as follows:

Patients were diagnosed with chronic myeloid leukemia in the chronic phase. 2. Patients only received imatinib as initial treatment. 3. Bone marrow specimens of patients can be obtained at the time of diagnosis and 3 months after imatinib treatment. 4. Response to Imatinib must be monitored according to cytogenetic and molecular criteria.

Patients who do not meet the inclusion criteria were excluded. Eighty-five patients (20 AML, 65 CML) and fifty-six health control were enrolled who were admitted to the First Affiliated Hospital of Zhejiang University (Hangzhou, Zhejiang province). The study was approved by the Ethics Committee of the First Affiliated Hospital, Zhejiang University College of Medicine. All patients signed the informed consent for the use of clinical data and the study was conducted in accordance with the Declaration of Helsinki.

Sixty-five CML-CP patients (21 females and 44 males) with a median age of 40 years (range, 15-68) and a median follow-up of 42 months (range, 4-74months) who were diagnosed and treated in the Department of Hematology, the First Affiliated Hospital of Zhejiang University from 2009 to 2013, were included in this retrospective study. All the patients have received imatinib 300-400 mg/d after being diagnosed. The characteristics of these patients are shown in Table 1.

### Definition of treatment response

Responses including complete cytogenetic response (CCyR), partial cytogenetic response (PCyR), major molecular response (MMR) and complete molecular response (CMR) were defined according to European Leukemia Net recommendations 15,16.

### Reagents and Antibodies

Imatinib was purchased from Selleckchem company (Houston, USA). After being dissolved in dimethyl sulfoxide (DMSO; Sigma-Aldrich, St. Louis, MO, USA), it was subpackaged in different concentrations and stored at -20℃. The following antibodies were used: anti-cofilin, anti-p-cofilin (Ser-3), and anti-GAPDH, which were all from Cell Signaling Technology (Beverly, MA, USA). Anti-Arp2, anti-Arp3 were from Abcam (Cambridge, UK).

### Cell Culture/Treatment with imatinib

The human cell line K562 (purchased from the National Collection of Authenticated Cell Cultures, Shanghai, China) were cultured in RPMI-1640 (Gibco, Billings, MT, USA), supplemented with 10% fetal bovine serum (FBS, Gibco Billings, MT, USA), 100 μg/ml penicillin, 100 U/ml streptomycin at 37°C in a suitable incubator with 5% CO_2_. Cells were treated with imatinib in different concentration for 24 hours until 50% confluency was achieved.

### Quantitative RT-PCR

Total RNA was extracted using TRIzol (Invitrogen, Carlsbad CA, USA). RT-PCR was conducted using the iQ SYBR Green Supermix and iCycler Real-time PCR Detection system (BioRad, Hercules, CA, USA) according to the manufacturer's instructions. The forward primer and reverse primer used to amplify CFL1 were GCGTAAGTCTTCAACGCCAGAG and TCGACAGTCTGGCCCACATC, respectively. Relative mRNA expression was normalized to the expression of glyceraldehyde-3-phosphate dehydrogenase (GAPDH) in each treatment and untreated control.

### Western blot analyses

Cells were lysed in 1x radioimmunoprecipitation assay (RIPA) buffer. Protein lysates were separated on SDS-PAGE and transferred onto PVDF membranes. Proteins were detected with primary antibodies at 1:1000 dilution and with HRP-conjugated secondary antibody at 1:5000 dilution (KPL, Baltimore, MD, USA). The results were analyzed using an ECL kit (Amersham, Little Chalfont, UK) and imagine lab software (Bio-Rad Laboratories, Hercules, CA, USA).

### G/F-actin Quantification

G-actin and F-actin were separated according to the manual of G-actin / F-actin In Vivo Assay Kit (Cytoskeleton, Inc. cat. #BK037). Briefly, harvest 1 x 107 cells by centrifugation at 1,000 x g for 2 minutes. Resuspend cell pellet in 500 µl of warm Lysis and F-actin Stabilization Buffer. Incubate lysates at 37°C for 10 minutes. Centrifuge at 100,000 x g, 37°C for 1h. The G-actin fraction is in the supernatant whereas the F-actin is in the pellet. Then, the samples of supernatant and pellet are run in an SDS-PAGE system, and actin is quantitated by western blot analysis.

### Statistical analysis

An independent sample t-test was used for mean comparison. The paired samples were compared by paired t-test, and the rates were compared by the χ^2^ test or Fisher's exact test. A logistic regression model was used for univariate and multivariate analysis. Variables were entered in multivariate analysis when *P* < 0.1. Two-tailed *P* < 0.05 was defined as the significance level. Statistical analyses were carried out using the SPSS version 13.0.

## Results

### Patients and baseline characteristics

All 65 CML patients included in this study received imatinib 400 mg/d after diagnosis. The expression of cofilin was evaluated at the time of diagnosis of CML and 3 months after starting imatinib therapy. All patients received routine monitoring according to ELN recommendation 16. The baseline data are given in Table 1.

### The expression of CFL1 in different patient groups

**Figure 1:**
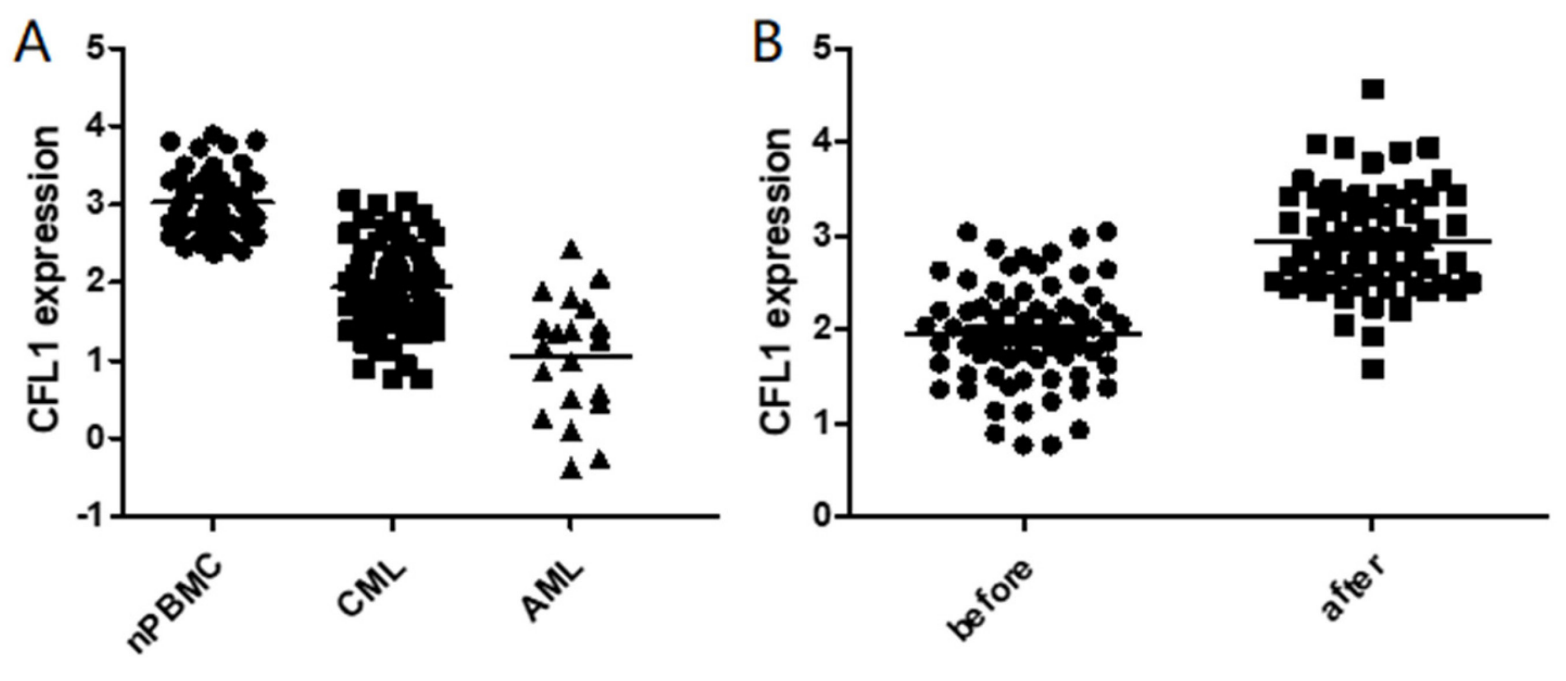
The expression of CFL1 in different patients. A: The expression of CFL1 in CML, AML and healthy control. B: The expression of CFL1 in CML-CP patients before and after imatinib therapy. The expression of CFL1 in bone marrow cells of new diagnosed CML, AML patients, CML-CP patients before and after 3 months imatinib therapy and in PBMC of healthy control were tested using Quantitative RT-PCR. Statistics performed t-test, p<0.05. (nPBMC: peripheral blood mononuclear cells from healthy control, CML: chronic myeloid leukemia, AML: acute myelocytic leukemia, before: before imatinib therapy, after: after imatinib therapy).

We initially analyzed CFL1 mRNA in each patient group. The median expression level of CFL1 was 2.98 (1.23-3.98) in 56 normal controls, 1.92 (0.77-3.05) in CML-CP patients and 1.22 (0.38-2.42) in AML patients, respectively. The frequency and extent of CFL1 expression among these three groups were analyzed by One-Way ANOVA (Table 3). The expression level of cofilin was lower in CML-CP patients than in healthy controls. The lowest expression level of cofilin was observed in AML patients (p < 0.05) (Figure 1A).

Furthermore, we evaluated the expression of CFL1 during imatinib therapy. When CML was diagnosed, the median expression level of cofilin was 1.92 (0.77-3.05), and it increased significantly after 3 months of imatinib treatment [2.94 (1.58-4.57), p<0.001] (Figure 1B). We further compared CFL1 expression level of each patient with 52 pared CML-CP patients, in which CFL1 was tested twice before and after 3 months imatinib therapy. In these CML patients, the expression level of CFL1 significantly increased from 1.86 (0.77-3.04) to 2.99 (1.58-4.57) after a 3-month imatinib therapy(p<0.001) (Supplementary Figure 1).

### The relationship between the CFL1 level and the response to imatinib

Next, we investigated the relationship between the CFL1 level and the response to imatinib. We defined We defined “high expression level of cofilin” as relative gene expression equal to or higher than the median level and “low expression” as relative gene expression lower than the median level. Patients in the cofilin 1 high expression group were significantly less responsive to imatinib compared to those in the low expression group at 6 months of treatment (Table 2).

To confirm if the cofilin expression level predicts the response to imatinib at early stages of treatment, we analyzed the cofilin expression and baseline BCR-ABL transcriptional level. The results showed no relationship between cofilin expression and BCR-ABL transcriptional level at baseline (R = -0.065, p = 0.608). To evaluate the relationship between CML disease itself and the expression of cofilin, we performed univariate and multivariate logistic regression and found WBC and PLT counts were independent factors influencing cofilin expression when CML was diagnosed (Supplementary Table 1,2). The potential correlation of the frequency and extent of CFL1 expression with CML biomarkers was shown in Table 4.

### Imatinib inhibits cofilin activity and upregulate F-actin forming

**Figure 2:**
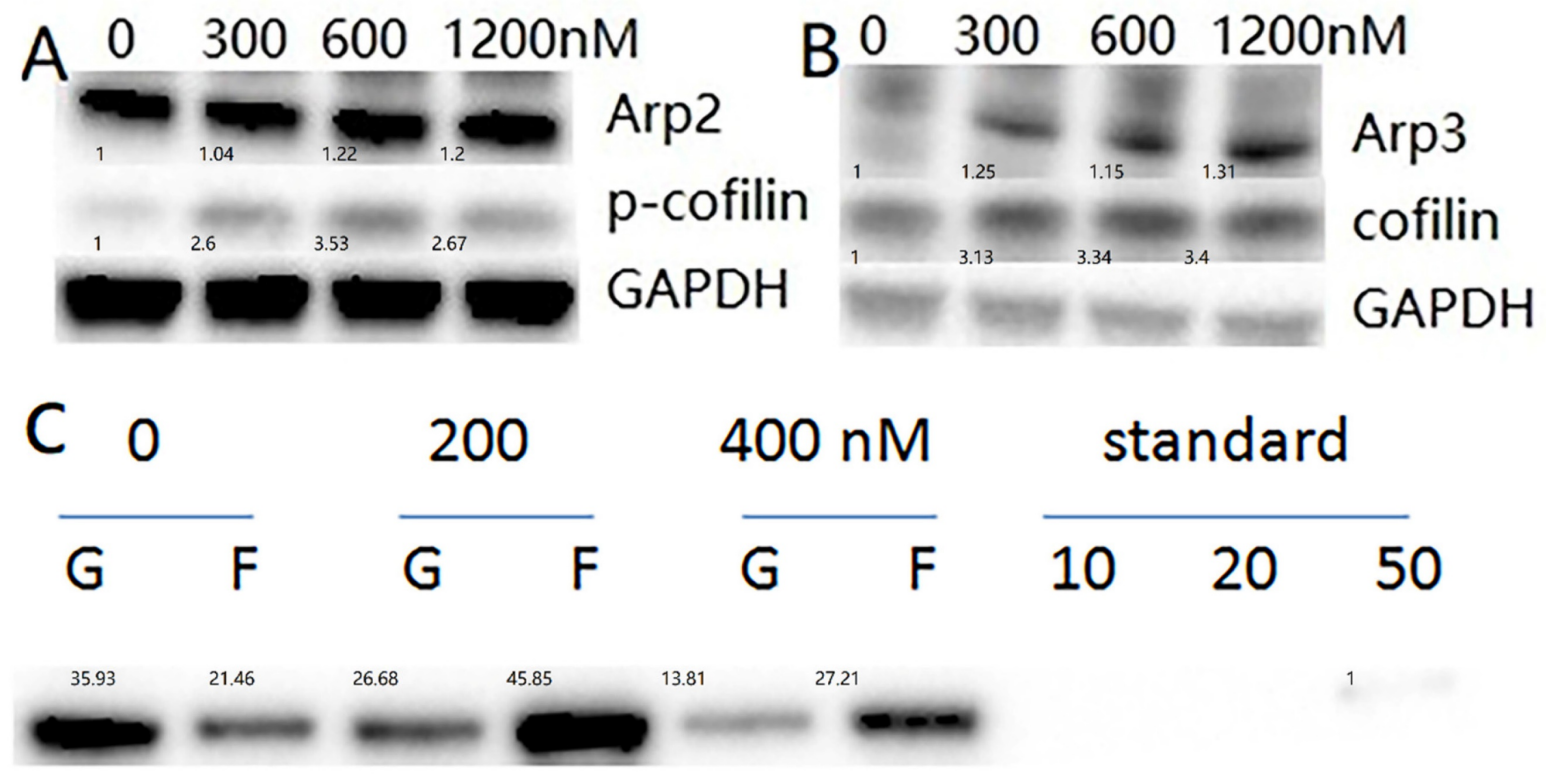
The expression levels of cofilin, p-cofilin, Arp2, Arp3 and F-actin percentage in K562 cells were increased after treatment with imatinib for 24 hours. A, B: The expression levels of cofilin, p-cofilin, Arp2, Arp3 in K562 cells. The expression levels of cofilin, p-cofilin, Arp2, Arp3 in K562 cells were tested using western blot. Imatinib concentration is 0,300nM,600nM,1200nM, for 24 hours. (nM: nmol/L). C: F-actin percentage. After imatinib treatment for 24 hours, G-actin and F-actin were separated and quantitated by western blot analysis in K562 cells. Imatinib concentration is 0,200nM,400nM. (Standard: standard sample, nM: nmol/L).

The results suggested that a lower expression level of cofilin in CML patients predicts better response to imatinib. However, it seems to contradict the fact that cofilin increased after imatinib therapy. To answer this question, we tested the protein level of cofilin and its inactivity state, p-cofilin, in CML cell line K562 cells. The results demonstrated that imatinib treatment for 24 hours increased cofilin and P-cofilin expression (Figure 2A,B). Inactivity state, p-cofilin, improved means cofilin ceiled function was inhibited.

The Arp2/3 complex is one of the major actin regulators, in which, Arp2 and Arp3 are structurally related to actin. It nucleates new actin filaments in the branching nucleation process 17. Our findings demonstrated an upregulation of Arp2 and Arp3 after a 24-hour imatinib treatment with K562 cells (Figure 2A,B).

When cofilin was phosphorylated at the ser3 site, the function of ceiled F-actin was inhibited. The level of F-actin /G-actin was then tested, and as expected, the F-actin percentage increased after imatinib treatment (Figure 2C). The results suggested that imatinib inhibited cofilin function, thus increasing the F-actin ratio.

## Discussion

Long-term survival is expected for the patients with CML who received TKIs. Therefore, careful management and evaluation of prognostic factors are of utmost importance for these patients. BCR/ABL expression after 3 months of TKI therapy is one of the well-known outcome predictors 5. However, not all CML patients are suitable for this test. It is imperative to identify independent factors for early prediction of response to imatinib and prognosis. Additionally, some factors, especially those related to CML, are essential to be explored.

Cofilin is one critical member of actin deploy family (ADF), which is involved in the biological activities of various human malignancies, including infiltration, metastasis, and chemotherapy resistance 18. With the function to ceil F-actin to G-actin, its activity was modified by phos-3ser/thr. Cofilin that is phosphorylated at Ser3 was long considered to be an inactive form without any biological function 19.

Overexpression of cofilin has been reported in human malignancies, and it is often associated with aggressive features or a poor prognosis. Cofilin is highly expressed in colorectal cancer and is associated with cancer progression and chemoresistance 20,21. Cofilin has also been implicated in the migration of colon cancer cells 22. In line with these observations, another study on colon cancer suggested that cofilin mediated epithelial-mesenchymal transition, cell migration and invasion in cancer cells 23. Analysis of pancreatic ductal carcinoma revealed an association between higher cofilin expression and poor differentiation, lymph node metastasis, surrounding invasion, later stage and shorter survival 24. Moreover, findings on prostate cancer revealed that cofilin expression increased significantly in metastatic tumors and coordinated responses to TGF-β, which was required for invasive cancer migration and metastasis 25.

However, little is known about the expression of cofilin in hematological diseases.

This study characterized the role of cofilin in CML and responses to imatinib. The results revealed significantly different cofilin expression in AML, CML and healthy control, indicating that mature myeloid cells had higher expression of cofilin. The finding contradicted the aforementioned results in solid cancers. It could be explained by different cell types with different actin dynamics and different migration potentials in blood cells.

To explore the involvement of cofilin in CML, multivariate analysis was performed on the baseline data of CML patients to identify the factors that influence cofilin expression. The results showed that WBC and PLT counts were independent factors for cofilin expression. WBC and PLT counts partly reveal the CML state, and we observed that higher WBC and PLT counts correlated with lower cofilin expression.

Our results showed higher CCR rates were achieved in the low expression group after a 6-month therapy, suggesting that lower cofilin expression seems to predict a better response to imatinib.

To further explore the role of cofilin during imatinib therapy, we tested its expression level before and after imatinib therapy. Our results show the expression of cofilin was unregulated significantly after therapy. More importantly, we observed a significant increase in cofilin expression after imatinib therapy in paired CML-CP patients. The results suggested that imatinib induced cofilin expression through certain pathways.

We further tested the expression of cofilin and p-cofilin, and found that the expression of both proteins significantly increased after imatinib treatment. A previous study using quantitative proteomic analysis revealed that only 10-30% cofilin is phosphorylated in myeloid tumor cell lines under standard conditions 26. Pharmacological inhibition of LIMK kinases increased its phosphorylation, and such a finding was partly consistent with our observations.

Phosphorylation of cofilin (P-cofilin) on a serine residue inhibits its binding to actin. Only dephosphorylated cofilin can carry out its severing or forming function 14,18. The role of cofilin in apoptosis is well established, i.e., activated cofilin can translocate to mitochondria and induce cell apoptosis 19,27,28. Actin is required in cell death processes. In leukemic cells, the translocation and accumulation of actin in the nuclear area are associated with apoptosis 29 and involved in chromatin remodeling during apoptosis in myeloid cell lines 30. To verify the state of actin during imatinib therapy, we tested the ratio of F-actin/G-actin and found that F-actin was upregulated after imatinib therapy. Further results showed that Arp2 and Arp3 were elevated as well. The Arp2/3 complex is required in branched actin network formation, and such a branched actin network promotes cell biogenesis and motility 31. These findings indicated that Imatinib increases the formation of branched F-actin.

Imatinib therapy may induce apoptosis by promoting CML cell movement, weakening the adhesion between CML cells and bone marrow endothelial cells, as well as reducing the sheltering effect of the bone marrow microenvironment on CML cells.

On the other hand, F-actin changes chromatin remodeling and cofilin localization to induce apoptosis. Yet, the exact mechanism needs to be further studied.

In summary, the results of this study provide a first insight into the role of cofilin in CML. CFL1 expression can serve as a robust prognostic predictor for CML patients. CML patients with lower CFL1 expression may show better responses to imatinib. Phosphorylation and inactivation of cofilin caused by imatinib impair its activity, leading to an increase of F-actin percentage, especially branched F-actin.

## Supplementary Material

Supplementary figures and tables.

## Figures and Tables

**Table 1:** Patient characteristics (N = 65)

Characteristics			No.	%
Age (y)				
Median			40	
Range			15-68	
Gender				
Male			44	67.7
Female			21	32.3
WBC (×10^9^/L)				
Median			154	
Range			7-740	
PLT (×10^9^/L)				
Median			407	
Range			70-2214	
Hb (g/L)				
Median			115	
Range			51-170	
Peripheral blood basophils (%)				
Median			2.9	
Range			0-12.2	
Peripheral blood eosinophils (%)				
Median			1.5	
Range			0-7	
Bone marrow blasts (%)				
Median			1.5	
Range			0-9	
Bone marrow basophils (%)				
Median			2.5	
Range			0-11	
Bone marrow eosinophils (%)				
Median			4	
Range			1-11	
Splenomegaly (thick, cm)				
Median			4	
Range			0-9.2	
*BCR/ABL1*^IS^ (%)				
Median			78	
Range			21-189	

*BCR/ABL1*^IS^ : The ratio of *BCR/ABL1* to *ABL1* on the international scale

**Table 2:** Treatment responses by CFL1 expression level at the time of CML diagnosis.

Groups	3 months BCR-ABL ≤ 10% N (%)	p	6 months MMR N (%)	p	6 months CCR N (%)	p
High	12 (37.5)	0.105	3 (9.4)	0.086	15 (46.9)	0.027^*^
Low	19 (57.6)		8 (25.8)		23 (74.2)	

* Indicates statistical significance

**Table 3:** The frequency and extent of CFL1 expression among three groups.

Group		N	Median (Min-Max)	Mean (95%CI)	F	p
CML-CP		65	1.92 (0.77-3.05)	1.04 (0.68-1.41)		
AML		20	1.22 (0.38-2.42)	1.96 (1.83-2.09)	108.959	<0.001
Health Control		56	2.98 (1.23-3.98)	2.99 (2.09-2.38)		

**Table 4:** The potential correlation of the frequency and extent of CFL1 expression with CML biomarkers

Variables		CFL1 Low (Mean,SD)	CFL1 High (Mean,SD)	t	p
Number		33	32		
WBC (×10^9^/L)		2.04 (179.91)	1.11 (101.56)	2.553	0.014
Hb (g/L)		99 (24.92)	112.19 (20.78)	-2.27	0.027
PLT(×10^9^/L)		417.63 (206.88)	564.12 (476.87)	41.99	0.118
Peripheral blood eosinophils (%)		2.799 (2.26)	2.457 (1.53)	32.71	0.571
Peripheral blood basophils (%)		3.273 (3.61)	3.457 (3.02)	-0.18	0.859
Bone marrow blasts (%)		3.224 (4.82)	1.885 (1.90)	1.295	0.205
Bone marrow eosinophils (%)		4.562 (2.02)	5.038 (2.31)	-0.77	0.444
Bone marrow basophils (%)		3.104 (2.97)	3.192 (3.12)	-0.1	0.919
*BCR/ABL1*^IS^ (%)		82.47 (36.98)	72.68 (32.98)	1.124	0.265

CFL1 Low: CFL1 Low expression CFL1 High: CFL1 High expression *BCR/ABL1*^IS^: The ratio of *BCR/ABL1* to *ABL1* on the international scale SD: Standard Deviation
