# Supplementary material for: CFL1 is Implicated in Chronic Myeloid Leukemia Response during Imatinib Therapy
Source: J Cancer. 2024 Mar 4;15(8):2424–30. doi: 10.7150/jca.92202 (PMC10937266; doi:10.7150/jca.92202)
Supplement: Supplementary file 1 — Supplementary figures and tables. [file jcav15p2424s1.pdf]

Supplementary Material

Supplementary Table1 Univariate analysis for cofilin expression before imatinib therapy.

variable	OR	p-value
Gender	0.458	0.123
Age	1.023	0.204
WBC ($\times 10^9$/L)	0.994	0.006*
Hb (g/L)	0.979	0.015*
PLT ($\times 10^9$/L)	0.997	0.065
Peripheral blood eosinophils (%)	0.913	0.12
Peripheral blood basophils (%)	0.991	0.079
Bone marrow blasts (%)	0.953	0.042*
Bone marrow eosinophils (%)	5.821	0.213
Bone marrow basophils (%)	4.943	0.291
BCR-ABL	0.992	0.271

* indicates statistical significance, OR: ods ratio

Supplementary Table2 Multivariate analysis for cofilin expression before imatinib therapy.

variable	β	OR	p value
WBC	-0.014	0.986	0.033*
PLT	-0.006	0.994	0.028*

* indicates statistical significance, OR: ods ratio

Supplementary Figure

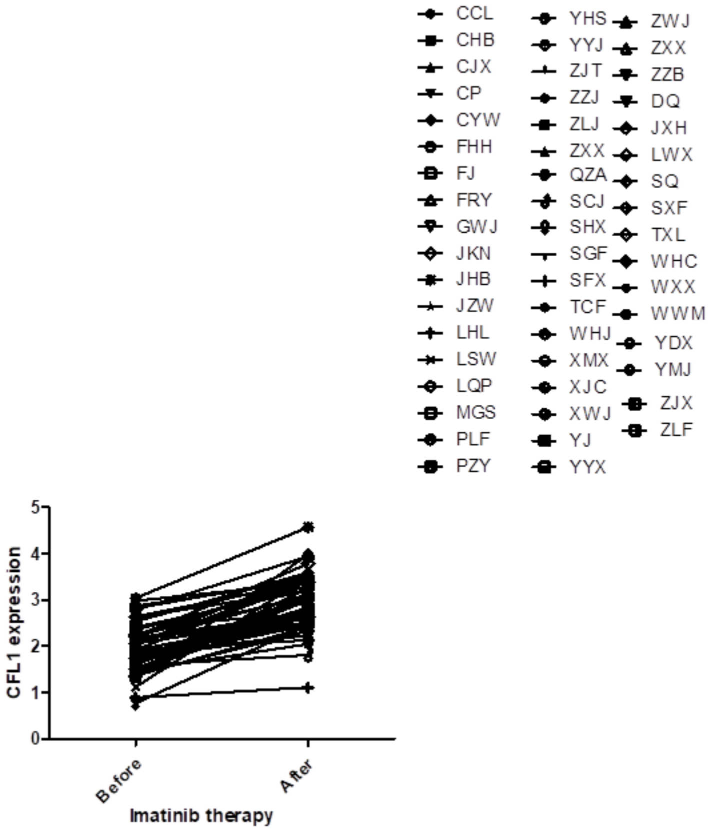

Supplementary Figure1

The CFL1 expression level of each patient was tested twice using Quantitative RT-PCR before and after 3 months imatinib therapy.

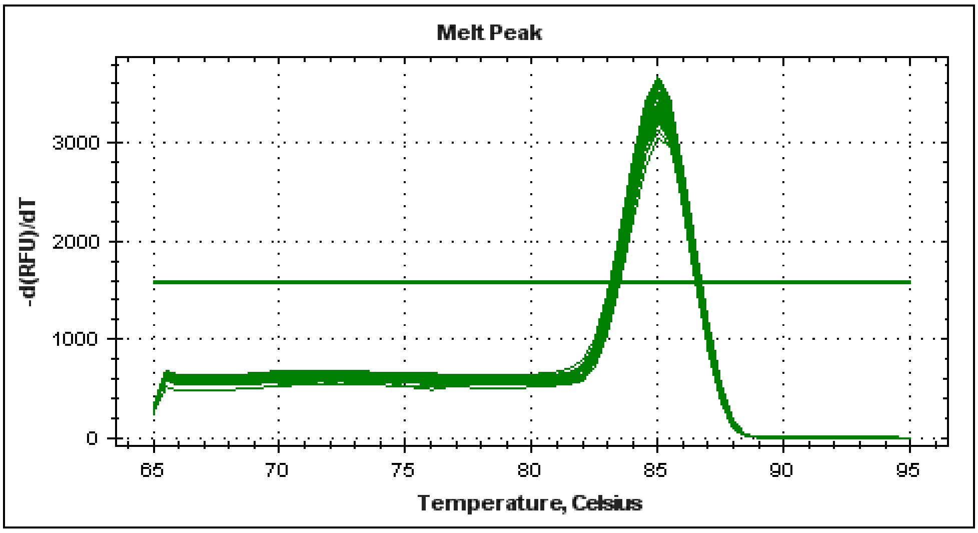

Supplementary Figure2 The real-time PCR dissociation curve of CFL1

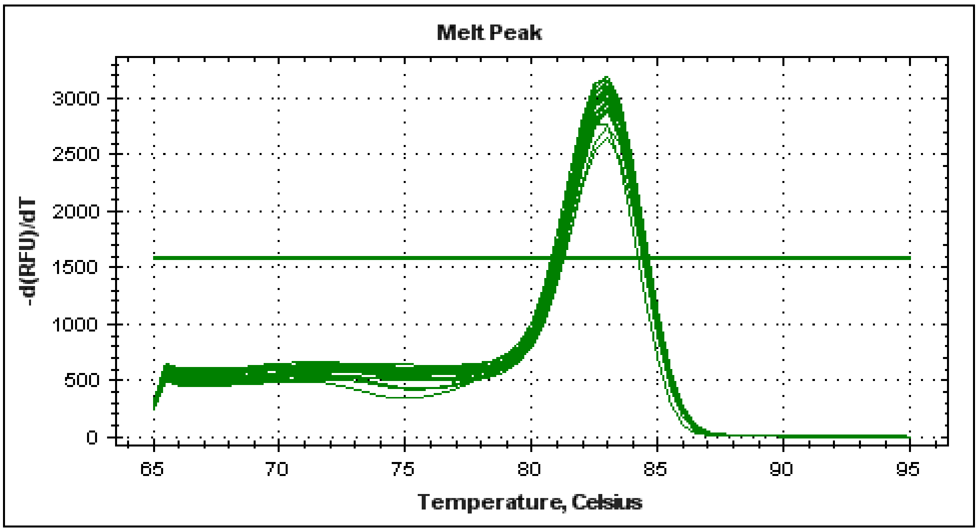

Supplementary Figure3 The real-time PCR dissociation curve of GAPDH

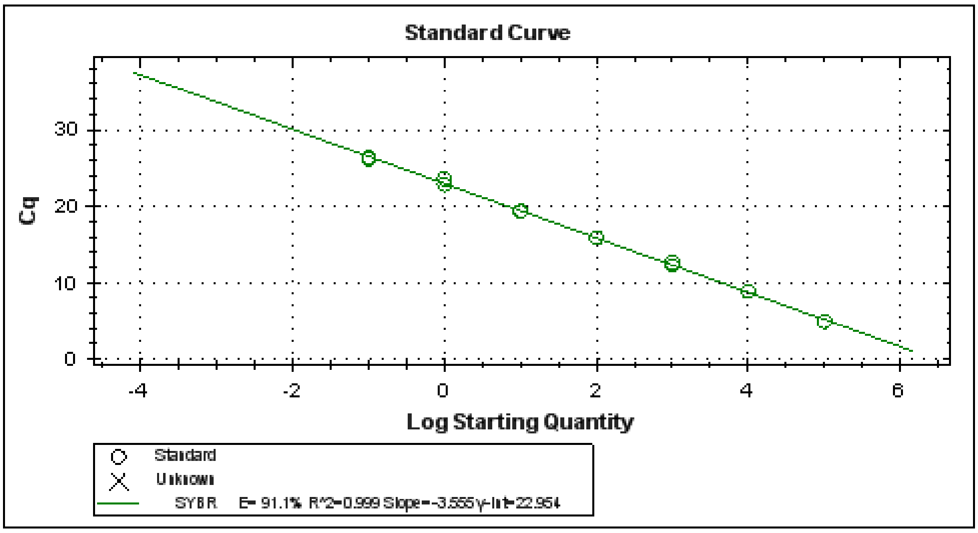

Supplementary Figure4 The real-time PCR amplification efficiency for CFL1

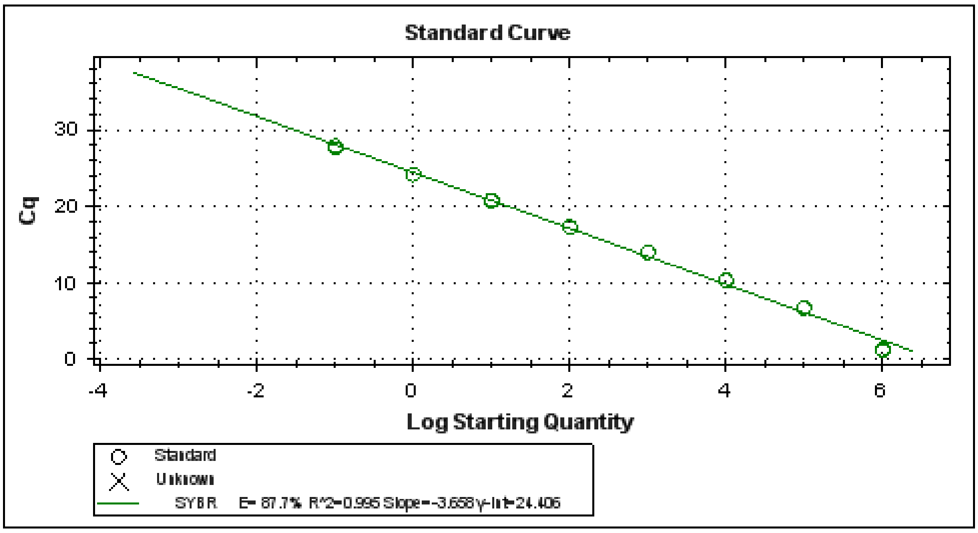

Supplementary Figure5 The real-time PCR amplification efficiency for GAPDH
